# NADPH- Diaphorase positive cardiac neurons in the atria of mice. A morphoquantitative study

**DOI:** 10.1186/1471-2202-7-10

**Published:** 2006-02-02

**Authors:** Laura Beatriz Mesiano Maifrino, Edson Aparecido Liberti, Patrícia Castelucci, Romeu Rodrigues De Souza

**Affiliations:** 1 Department of Anatomy, Institute of Biomedical Sciences, São Paulo University, São Paulo, Brasil; 2 Dante Pazzanese Institute of Cardiology, São Paulo, Brasil; 3 Department of Anatomy, São Judas Tadeu University, São Paulo, Brasil

## Abstract

**Background:**

The present study was conducted to determine the location, the morphology and distribution of NADPH-diaphorase positive neurons in the cardiac nerve plexus of the atria of mice (ASn). This plexus lies over the muscular layer of the atria, dorsal to the muscle itself, in the connective tissue of the subepicardium. NADPH- diaphorase staining was performed on whole-mount preparations of the atria mice. For descriptive purposes, all data are presented as means ± SEM.

**Results:**

The majority of the NADPH-diaphorase positive neurons were observed in the ganglia of the plexus. A few single neurons were also observed. The number of NADPH-d positive neurons was 57 ± 4 (ranging from 39 to 79 neurons). The ganglion neurons were located in 3 distinct groups: (1) in the region situated cranial to the pulmonary veins, (2) caudally to the pulmonary veins, and (3) in the atrial groove. The largest group of neurons was located cranially to the pulmonary veins (66.7%). Three morphological types of NADPH-diaphorase neurons could be distinguished on the basis of their shape: unipolar cells, bipolar cells and cells with three processes (multipolar cells). The unipolar neurons predominated (78.9%), whereas the multipolar were encountered less frequently (5,3%). The sizes (area of maximal cell profile) of the neurons ranged from about 90 μm^2^to about 220 μm^2^. Morphometrically, the three types of neurons were similar and there were no significant differences in their sizes. The total number of cardiac neurons (obtained by staining the neurons with NADH-diaphorase method) was 530 ± 23. Therefore, the NADPH-diaphorase positive neurons of the heart represent 10% of the number of cardiac neurons stained by NADH.

**Conclusion:**

The obtained data have shown that the NADPH-d positive neurons in the cardiac plexus of the atria of mice are morphologically different, and therefore, it is possible that the function of the neurons may also be different.

## Background

The heart of mammals is innervated by nerve fibers from the vagus and the sympathetic chain. In addition, a group of neurons situated at the dorsal surface of the atria contribute to the innervation of the heart. These neurons play an important role in the regulation of cardiac output during rest, exercise and cardiovascular disease [1].

Recent evidences suggest that nitric oxide (NO), acts as a neurotransmitter in both the central [2,3] and peripheral nervous system [4-6]. Nitric oxide is synthesized by the enzyme nitric oxide synthase (NOS) in a reaction that converts the amino acid arginine to citruline. It has become apparent that histochemical techniques to demonstrate NADPH- diaphorase enzymatic activity detect the presence of NOS in neurons [7]. Therefore, NADPH-d histochemistry is used to label NOS- containing neurons in the nervous system [8-11]. Previous studies have demonstrated the presence of NADPH-diaphorase positive neurons in the cardiac ganglia of the guinea-pig [12,13] and rat [7]. All cardiac neurons contained immunoreactivity to choline acetyl transferase, and two smaller subpopulations were immunoreactive to calbindin or NOS [14]. It was also demonstrated that 14.4% of cardiac nerve cell bodies of the mouse heart contain NOS [15]. The method of dye injection has permitted to establish that the morphology of neurons in autonomic ganglia bears a relation to their electrophysiological and histochemical properties [16-19]. In addition, physiological studies on the NADPH-d positive neurons of the cardiac plexus in mammals have been frustrated because nothing is known about the topography of these neurons [20]. Therefore, the present investigation was carried out to examine the location, morphology, distribution and the number and sizes of the NADPH-diaphorase positive cardiac neurons in the atria of mouse on whole-mount preparations. Our data show the existence of three types of neurons, based on their morphology and that these cells were located in 3 topographical groups.

## Results

### NADPH diaphorase positive cardiac neurons

**Figure 1:**
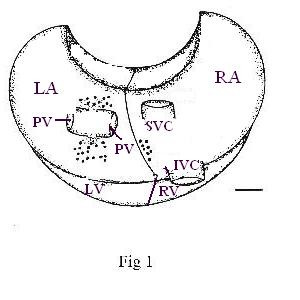
Diagram of epicardial surface of right and left atria of mouse heart viewed from their cranial aspect showing the 3 major regions in which NADPH-diaphorase positive neurons were located. The neurons are represented by dots. La= left atrium; Ra = right atrium; Pv = pulmonary veins; Svc = superior vena cava; Ivc = inferior vena cava; Lv = left ventricle; Rv = right ventricle. Calibration bar: 0.1 mm.

The location of the NADPH-diaphorase positive neurons is shown in the diagram of Fig. 1, in which they were represented by dots. In general, 3 groups of neurons were recognized. The first group of neurons was in the region situated cranial to the pulmonary veins, the second group of neurons was located caudally to the pulmonary veins and finally, the third group of neurons was located in the interatrial groove.

The number of NADPH-diaphorase positive neurons was obtained in laminae from 5 mice. This number ranged from 39 to 79 neurons. The average number of neurons was 57 ± 4. The largest group of NADPH-diaphorase positive neurons was located cranially to the pulmonary veins (66.7%). The neurons located caudally to the pulmonary veins formed 26.3% of the stained neurons. The neurons located in the interatrial groove were the fewest in number (7.0%).

**Figure 2:**
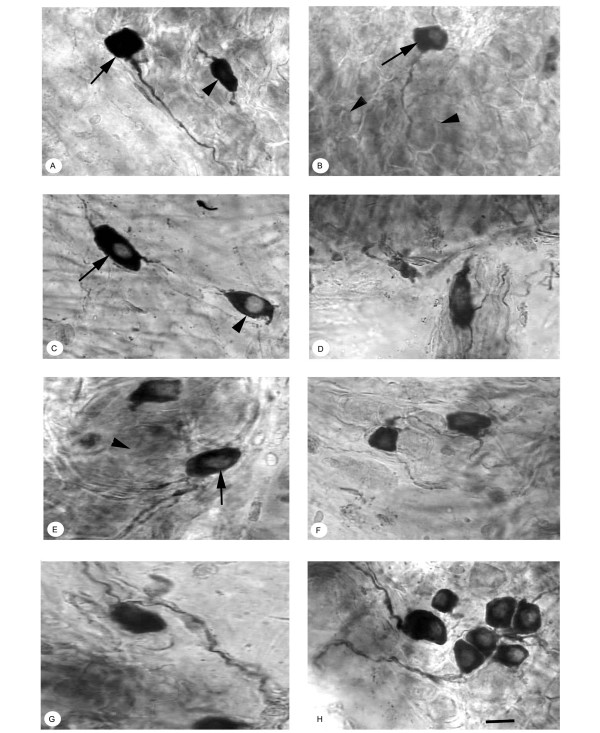
NADPH-diaphorase positive cardiac neurons of the mouse atria: (A) One of the NADPH-diaphorase positive neurons is unipolar (arrow) and the other is bipolar (arrowhead). (B) Note the unipolar NADPH-diaphorase positive neuron (arrow) and the nonreactive neurons (arrowheads). (C) An ovoid bipolar neuron with two short processes (arrow), and one multipolar neuron with three short processes (arrowhead). The neurons are pear-shaped with a round or oval nucleus. (D) A multipolar neuron with three processes. (E) A ganglion stained for NADPH-diaphorase. The ganglion contains both NADPH-diaphorase positive neurons (arrow) and nonreactive perikarya (arrowhead). (F) The two NADPH-diaphorase positive neurons are unipolar neurons. (G) A single NADPH-diaphorase positive neuron with two large processes. (H) Cardiac ganglion showing a cluster of unipolar NADPH-diaphorase positive neurons. Calibration bar: 20 μm.

The great majority of NADPH-diaphorase positive neurons were found in ganglia of the cardiac plexus. Each ganglion usually contained 1–7 NADPH-diaphorase positive neurons (Fig. 2H). Single NADPH-diaphorase positive neurons were occasionally found (Fig. 2B, G). There was a small variation in the intensity of the staining of neurons.

Three types of neurons could be distinguished on the basis of their shapes: cells with one labeled process (unipolar cells) (Fig. 2A, B, E, F, H); cells with two short processes (bipolar cells) (Fig. 2A, C, G) and cells with three short or long processes (multipolar cells) (Fig. 2C, D). The contours of unipolar cell bodies were of an irregular round or oval shape. The long processes of these types of neurons extended from the hillock over a few microscopic fields. In the great majority of them, the process was traced only within the ganglion. The contours of the bipolar neurons bodies were oval in shape. The processes of these neurons were traced only within the ganglion, with no axon distinguishable. The contours of the multipolar neurons, as well as of the bipolar ones, were oval in shape. One multipolar neuron had 3 short processes and one, had two short and one long process.

As to the location of the three types of neurons, cranially to the pulmonary veins, there were 32 unipolar neurons, 5 bipolar neurons and 1 multipolar neuron (n = 38). Caudally to the pulmonary veins there were 11 unipolar neurons, 3 bipolar neurons and 1 multipolar neuron (n = 15). Finally, in the interatrial groove there were 2 unipolar neurons, 1 bipolar neuron and 1 multipolar neuron (n = 4) (Table 1). The unipolar neurons were clearly predominant in the three regions.

**Figure 3:**
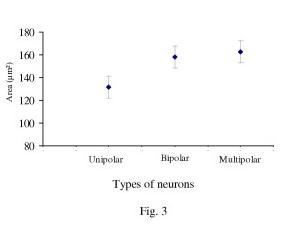
Relation between mean, standard error of mean and deviation values of sizes (μm^2^) of the three types of NADPH-positive cardiac neurons (unipolar, bipolar and multipolar) of the mouse atria.

The size of each type of NADPH-diaphorase positive neurons was assessed (as cell profile area) in the whole-mount preparations used for cell counts. Sizes of the three types of neurons stained for NADPH-diaphorase method, ranged from about 90 μm^2 ^to about 220 μm^2^. The mean size of each type of neuron was obtained and the data from the three groups were tested for significance using the one-way ANOVA (Fig. 3). The results showed that there were no significant differences between the sizes of the three types of neurons.

### NADH diaphorase positive cardiac neurons

Examination of the NADH-stained preparations revealed that the mean number of the neurons in the atrial preparations was 530 ± 23, n = 5). Comparing the number of NADPH-diaphorase positive neurons to the NADH positive cardiac neurons, we concluded that the percentage of NADPH-diaphorase positive to cardiac neurons in this study was 10%.

## Discussion

Although the NADPH- positive intracardiac neurons of many animals have been investigated, a structural organization and topography of these neurons in mice have not been described. Therefore, in the present study we have attempted to describe exactly the location, the morphology, the number and the sizes of NADPH-diaphorase positive cardiac neurons in the atria of mice.

At present, many studies are carried out using NADPH-diaphorase reaction with nitro blue tetrazolium [7,8,21-26]. Applying the NADPH-diaphorase technique and immunohistochemistry with NOS antiserum, it was examined the occurrence of NOS in nerve fibers and neurons of the rat and guinea-pig heart [12]. According to these authors, NOS immunoreactivity and NADPH-diaphorase technique showed identical results. However, some authors [27] encountered a significant difference in the number of NADPH-d positive and NOS immunoreactive neurons in the urinary bladder of guinea pig. The authors suggest that the localization of one enzyme does not necessary reflect the presence of the other. When comparing the results achieved by NOS-immunocytochemistry and those by NADPHd-histochemistry, the distribution patterns of positive nerve-cells and fibers were apparently similar. A combination of both methods revealed complete colocalization of NOS and NADPHd in neuronal perikarya and fibres [9].

The use of whole mount preparations presents the intracardiac plexus as a monolayer of nerve cells, and it has therefore been possible, in parallel with neuron location and counts, to make measurements of neuron somata of unsectioned nerve cells. However the two techniques used in this investigation do not allow seeing terminals in the intrinsic cardiac ganglia around intrinsic neurons.

Our data about the location and distribution of NADPH-positive cardiac neurons showed the presence of three major groups of ganglion cells, one cranially to the pulmonary veins, one caudally to the pulmonary veins and one dorsal to the interatrial groove. The right atrial free wall, atrial appendages and trunk of great vessels were devoid of cardiac NADPH-diaphorase positive neurons. However, in the guinea-pig heart, the largest populations of NOS-immunoreactive neurons were distributed in right atrium, septum and left atrium [13,28]. The distribution of NADPH-diaphorase positive neurons in specific ganglia that we observed in this study indicates that they selectively project to and thus control specific cardiac neurons.

NADPH-diaphorase cardiac ganglia of mouse contained three morphological types of neurons. Neurons in one group, were unipolar. The second set of neurons, were bipolar and the third set of neurons were multipolar. The unipolar predominated (78.9 % of the stained neurons), whereas the bipolar and multipolar were encountered less frequently (15.8 % and 5.3 % of the sampled neurons, respectively). The three types of NADPH-positive neurons were encountered in the three regions of the mouse atria. These results suggest that, at least for the mouse's heart, the morphology of neurons is not conditioned by their location. However, the importance of the location has been showed in a few physiological studies, in which it was demonstrated that the electrophysiological properties of the intracardiac neurons are related to their different location in canine heart [29]. The types of neurons observed in the present work are similar to that lying in the nerve plexus of the cardiac hilum of the guinea pig [20,30]. The relatively great abundance of unipolar neurons we have encountered is also similar to the results obtained for the neurons of the nerve plexus of the cardiac hilum of rats and guinea pigs [20]. The existence of cardiac neurons with no dendritic arborization, in the guinea pig has been confirmed by labeling them intracellularly with neurobiotin [31]. It is possible that some of the neurons observed in the present work provide postganglionic innervation to the heart or possibly some may function as interneurons to ganglion cells in other cardiac ganglia or in the same ganglion. In fact, some studies of the NADPH-diaphorase cardiac neurons indicated that a number of neurons in the rat heart and in the guinea-pig heart are NOS- immunoreactive and labeled neuronal processes are associated with cardiac ganglia, nodal tissues, the coronary vasculature and myocardium [25,32]. Therefore it is very likely that NO acts as a neuronal messenger of cardiac nerves innervating vascular smooth muscle, intrinsic neurons, nodal tissues and the myocardium [12].

The results of several studies support strongly the hypothesis that NO liberated from neuronal sources has an important facilitatory action on the vagal control of the heart [33-35]. The results of other study showed that NO plays a stimulatory role in mediating vagal neurotransmission and vagal modulation of sympathetic effects and an inhibitory role in mediating sympathetic neurotransmission [36]. In addition, prior pharmacological studies in guinea pigs suggest that NO facilitates the negative chronotropic effects of vagal stimulation [34,37-40]. In a recent paper, [15] it was investigated whether enhanced cardiac vagal responsiveness elicited by exercise training is dependent on neuronal nitric oxide synthase. The results suggest that the changes in vagal responsiveness resulted from presynaptic facilitation of neurotransmission, and that NOS appears to be a key protein in generating the cardiac vagal gain of function elicited by exercise training.

It was suggested that the mechanism of action of NO may be facilitation of acetylcholine release, either at the preganglionic-postganglionic or at postganglionic-muscle synapse [34,40].

Finally, examining cardiac effects induced by electrical stimulation of discrete loci within the ganglionated plexus located on canine atria it was concluded that atrial ganglia contain not only efferent parasympathetic, efferent sympathetic but also afferent neurons [41]. These atrial neurons received afferent inputs from sensory neurites in both ventricles that were responsive to local mechanical stimuli and the nitric oxide donor nitroprusside [42]. Therefore, it is possible that, some of these NADPH-d neurons are pain conducting afferent neurons.

The number of NADPH-diaphorase positive neurons in the cardiac plexus of mice averages 57. This number is relatively small when compared with the number of NADH-diaphorase positive neurons in the atria (530). In the other words, according to the present results, the NADPH- diaphorase positive neurons represents only 10% of the number of NADH positive cardiac neurons. However, [15] demonstrated the presence of 14.4% of NO-positive neurons in the mouse atria. On the other hand, the percentage of NADPH-diaphorase positive neurons to myenteric neurons in the rat stomach was 20% [43]. The difference suggests the possibility that the local physiological requirement of the organs may require different amounts of NO activity [43].

In terms of neuron size the NADPH-diaphorase positive cardiac neurons do not form a uniform population. They range in the area of their profile from about 90 μm^2 ^to about 220 μm^2^. The morphometric analysis of the NADPH-positive neuron types of the cardiac plexus has not showed statistically significant differences in somata parameters. A lack of the morphometric differences between distinct neuron types is known from various studies of the cardiac plexus [20,44]. An elucidation of such a pattern in morphometry of neurons confirms the fact that mostly morphometry of the somata does not serve as a diagnostic feature in the neuron classifications [20]. Neuronal sizes vary less in the cardiac than in the enteric ganglia [45] and resemble in this respect the ganglia of the mouse tracheae [46]. It is not clear at the present whether the variation in size within a population of neurons reflects differences in the extent of their territory of innervation [44]. That the volume of the tissue innervated influences the size of the intrinsic neurons is confirmed by the experiments of intestinal hypertrophy. A massive enlargement of the musculature on the oral side of a partial obstruction is accompanied by a marked neuronal hypertrophy, the average area of the cell profile increasing by about 100% [45].

## Conclusion

In summary, the present results shows that the cardiac ganglia present in the atria of mice are composed of about 10% of NADPH-d positive neurons. Morphologically, these neurons are of three types: unipolar, bipolar and multipolar. The majority of them have their processes confined within the ganglion and may possibly be the intraganglionically active neurons or interneurons. The existence of different types of neurons in the cardiac plexus of mice is an indication that they possibly have different functions. These neurons might mediate complex integration and cardiac reflex.

## Methods

### Animals

Ten, young, male, isogenic mouse, ASn, weighing 15–18 g were used. Mice were housed singly in cages and maintained under standard conditions at 21°C, with 12 h light dark cycle, and all water and food ad libitum.

Each animal was killed with an overdose of ether. The chest was opened, the heart removed and the atria were separated from the ventricles and dissected in order to remove excess of fat and connective tissue from their external surface. The study was conducted according to current legislation on animal experiments of the Biomedical Science Institute of the University of São Paulo.

### NADPH- Diaphorase histochemistry

Immediately after the atria were removed from the body and dissected, they were washed in saline. Five atria were then fixed in 4% paraformaldehyde in phosphate buffered saline (PBS) pH 7.2, at 4°C for 30 minutes. After 2 × 10 minutes rinses in PBS at room temperature, the atria were incubated in the following solution for the demonstration of NADPH-d for 60 minutes at 37°C, in the dark and with continuous agitation: β- NADPH reduced form (Sigma) 0.1 mg/ml., nitro blue tetrazolium (Sigma) 0.5 mg/ml in PBS containing 0.2% triton X-100 [20]. The interatrial septum was removed and the whole mount preparations of the atria were mounted in glycerol for microscopic examination. The formazan deposits filling the cell bodies and processes, and their absence in the cell nuclei identified the NADPH-diaphorase positive neurons. Using this method we were able to distinguish between the different types of neurons based on their morphology. For the neurotopographical investigations, the neurons were visualized on the stained whole mount preparations and were surveyed microscopically at magnification 50x.

### Morphometry

The numbers of NADH-diaphorase and of each morphological type of NADPH-diaphorase positive neurons were obtained directly at the microscope by using a 40 × objective. Neuron somata measurements were made on all nerve cell types present in each laminar preparation. The microscope image was projected to a monitor and neuron somata were outlined and measured on a digitizing tablet interfaced with a Kontron-300 image analyzer (Kontron Elektronik, Germany). The statistical significance of the difference between the means was calculated through a one-way ANOVA. Significance level was determined at *p *< 0.05.

### NADH Diaphorase histochemistry

To estimate the total number of cardiac neurons, 5 whole mount preparations of the atria were stained by a histochemical technique [22]. After the intraperitonial injection with Pentobarbital sodium (0.1 ml/100 g body wt) to anesthetize the animals, these were perfused with Krebs solution. Once the thoracic cavity was opened, the heart-lung blocks were isolated and, subsequently, the atria separated from the ventricles and by careful dissection with stereoscopic microscope, the subepicardium fatty tissue was removed. The atria were left in a Krebs solution for 30 minutes and afterwards placed in Triton-X solution (0.3% in Krebs solution) for 10 minutes. They became slightly agitated and were then washed four times for 3 minutes in the Krebs solution. Then the pieces were kept in the solution for incubation (25 parts of stock solution 0.5 mg/ml of Nitroblue Tetrazolium in distilled H_2_O, 25 parts of 0.1 M phosphate buffer pH 7.3, 50 parts of distilled water and 0.5 mg/ml of b-NADH diaphorase for 45 minutes, with the reaction monitored through a stereoscopic microscope. Once this time was over, the reaction was interrupted by the fixation of the material in buffered neutral 10% formalin solution (0.1 M sodium phosphate buffer, pH 7.3). The pieces were reduced to whole mount preparations through dissection under stereoscopic microscope and immediately after, washed in distilled water, and mounted in buffered glycerin jelly (0.2 M sodium phosphate buffer, pH 7.3) at 70° for light microscope examination.

## Authors' contributions

LBMM conceived and designed the study, performed acquisition, analysis and interpretation of data and drafted the manuscript. EAL conceived and designed the study. PC conceived and designed the study. RRS conceived and designed the study, analysis and interpretation of data and have given final approval of version to be published. All authors read and approved the manuscript.

## Figures and Tables

**Table 1:** Distribution of the NADPH- positive cardiac neurons of the mouse atria (means), according to their morphologic types.

Nerve cell group location	Unipolar	Bipolar	Multipolar	Total number of Neurons	%
Cranially to the pulmonary veins	32	5	1	38	66,7
Caudally to the pulmonary veins	11	3	1	15	26,3
In the interatrial groove	2	1	1	4	7,0
Total number of neurons (n = 5)	45	9	3	57	

%	78.9	15.8	5.3	100	
